# Knowledge, Attitudes and Practices of Chagas a Neglected Tropical Disease in Rural Communities of the Colombian Caribbean, CHAGCOV Study

**DOI:** 10.1007/s11686-024-00833-y

**Published:** 2024-04-09

**Authors:** Margarita M Ochoa-Diaz, Daniela Orozco-Garcia, Ronald S. Fernandez-Vasquez, Melisa Eyes-Escalante

**Affiliations:** 1 School of Medicine, Universidad del Sinú Seccional Cartagena, 130001 Cartagena, Colombia; 2 Research Group GIBACUS, Tropical Medicine, Cartagena, Colombia; 3 School of Biology, Tropical Medicine Doctorate, Universidad del Atlántico, Barranquilla, Colombia; 4 Research Group Biodiversidad del Caribe Colombiano, Barranquilla, Colombia

**Keywords:** Chagas Disease, Neglected Tropical Disease, Sustainable Development Goals, Colombia

## Abstract

**Purpose:**

Chagas disease (CD) a Neglected Tropical Diseases is an important public health issue in countries where is still endemic, included in the Sustainable Development Goals (SDG). Traditionally restricted to rural areas with diverse routes of transmissions from vectorial to oral with acute manifestations but being more common diagnosed in chronic stages. The aim of this investigation was to characterize the Knowledge, Attitudes and Practices (KAP) related to Chagas disease (CD) in two rural settlements of the Colombian Caribbean with previous records of the disease and/or the parasite.

**Methods:**

A cross-sectional descriptive study was made in two rural settlements in Colombia and surveillance instrument was developed to measure Knowledge, Attitudes and Practices (KAP) related to Chagas disease (CD).

**Results:**

In a population with > 60% women and access to social security around 66.5%; 81,6% were homeowners with access to water and electricity > 90% but only 9% of sewerage. The level of knowledge about CD was around 62% but lack of specificity about comprehension of transmission routes (74,6%), and symptoms (85,3%) were found; concluding that 86% of the surveyed sample had very poor level of knowledge about the disease despite preventive campaigns carried out in the two communities studied.

**Conclusions:**

Despite of a low frequency of CD in this Caribbean areas, the presence of vector, risk factors plus poor level of knowledge about the disease justify that public health intervention strategies should be implemented and monitored over time to maintain uninterrupted surveillance of Chagas Disease.

**Supplementary Information:**

The online version contains supplementary material available at 10.1007/s11686-024-00833-y.

## Introduction

Chagas disease (CD) is considered an important public health issue in countries where is still endemic, additionally belongs to the Sustainable Development Goals (SDG), specifically SDG number 3 with regard to the health component [1]. 

CD or American Trypanosomiasis is an anthropozoonoses belonging to the group of Neglected Tropical Diseases (NTD) that annually infects approximately 7 million people and remains endemic in 21 Latin American countries [2, 3]. This infection, initially was restricted to rural areas and due to intense urbanization and migratory phenomena, has increasingly taken over urbanized scenarios. The routes of transmission are diverse, ranging from vectorial transmission through contact with feces of the vector of the Triatominae genus infected by the *Trypanosoma cruzi (T. cruzi)* parasite; ingestion of food contaminated with feces of the infected vector; through contaminated transfusions; via congenital transmission or through transplants not screened for infection. Thus, if infection occurs, it can have manifestations which can be acute or chronic, sometimes going unnoticed by the health system [3–5] The purpose of the following research was to characterize the Knowledge, Attitudes and Practices (KAP) in relation to Chagas disease in two rural settlements of the Colombian Caribbean with previous records of the disease and/or the parasite [6]. 

## Materials and methods

This was a cross-sectional descriptive study. The research project also named “CHAGCOV project” because was performed during post-COVID took place in two rural settlements in the Colombian Caribbean region. The first one was a village in the Department of Atlántico named Corrales de San Luis (coordinates 10º52 × 27´´N74º58 × 43´´O), described by local research activities as a site of triatomine and *T. cruzi* circulation with no reports of positive cases of Chagas disease at the time of this research. Similarly, the municipality of Villanueva (coordinates 10º26 × 39´´N75º16 × 29´O) in the Department of Bolivar, was investigated, Villanueva had previous reports of orally transmitted Chagas cases [6, 7]. 

The target population age of the study was people over one (1) year old, being resident of the selected municipality. All the data was collected during 2021by an administered questionary to every recruited individual. All the dwellings of the selected populations were surveyed. Once the aim of the study was explained to the members of the house and after signing the informed consent form (individual or the responsible adult), the data collection instrument (administered questionary) was applied by the research member, remembering that the parent should answer the questions in the case o < 8 years old kids The structure of the instrument (survey) consisted initially of sociodemographic data (age, origin, sex, schooling, social security, occupation), variables related to household composition (number of inhabitants, basic sanitation); housing structure (construction material, use of repellents, domestic animals); knowledge of Chagas disease (7 questions on the disease, 7 questions on the vector); and the question of identification of positive cases in the community. Finally, the level of knowledge was rated as “Excellent”, “Fair” or Very Poor according to the responses obtained and the score applied of the answers.

All information was collected by health professionals. Informed consent was obtained for all patients who agreed to participate. All the information collected was recorded in an electronic database and analyzed with Epi-Info v. 7.1 CDC® software. The information collected was analyzed by descriptive statistics according to its nature, using statistical significance for all analyses as ≤ 0,05. Quantitative variableswill be explained by measures of central tendency (mean, media, mode). Parametric and no parametric test will be performed according the nature of the variables and its distribution (normality).

## Results

### Sociodemographic Characterization of the Population

A total of 272 inhabitants of both municipalities were included. In general, 65.8% of those surveyed were women, with an average age of 27 (interquartile range -RIC- 17–39) years, 92.7% were Colombian nationals and predominantly of socioeconomic stratum 1 (98.9%). In terms of access to social security, 66.5% belonged to a state subsidized regime and 7% had no access to the health system; with regard to occupation, 40.8% were engaged in housework, followed by school work (29.8%) and only 2.9% in agricultural work. In respect of schooling, 39.3% had secondary schooling and 7.4% had none. The demographic composition of the study population was distributed similarly in both municipalities (see Table 1).

**Table 1:** Sociodemographic distribution of the population studied, Corrales de San Luis, Atlántico and Villanueva (Bolívar)

	Total *N* = 272 n (%)	Corrales de San Luis *N* = 132 n (%)	Villanueva *N* = 140 n (%)	p Value
Sex				
F	**179 (65.8)**	**76 (57.6)**	**103(73.6)**	0,005
M	93 (34.2)	56 (42.4)	37(26.4)	
Age Me (RIC)	**27 (17–39)**	**27 (17–40)**	**27 (17–38)**	0,328
Nacionality				
Colombian	**252 (92.7)**	**130 (98.5)**	**122(87.4)**	0.174
Venezuelan	20 (7.3)	2 (1.5)	18(12.9)	
Stratum				
1	**269 (98.9)**	**129 (97.7)**	**140(100)**	0,112
2	3 (1.1)	3 (2.3)	0 (0.0)	
Social Health Regime (SHR)				
Subsidized	**181 (66.5)**	**96(72.7)**	**85(60.7)**	0,036
Contributory	70 (25.7)	28 (21.2)	42 (30.0)	0,097
None	**19 (7.0)**	**7(5.3)**	**12(8.6**)	0.290
No Data (ND)	2 (0.7)	1 (0.8)	1(0.7)	0,966
Occupation				
Home	**111 (40.8)**	**54 (40.9)**	**57(40.7)**	0,974
Student	**81 (29.8)**	38 (28.8)	43 (30.7)	0.728
Employee	28 (10.3)	8 (6.1)	20(14.3)	0,026
Other	28 (10.3)	14 (10.6)	13 (9.3)	0.715
Merchant	15 (5.5)	9 (6.8)	6 (4.3)	0,360
Agricultural work	8 (2.9)	7 (5.3)	1 (0.7)	0,032
Minor	3 (1.1)	2 (1.5)	1(0.7)	0.613
Pensionary	1 (0.4)	1 (0.8)	0 (0.0)	0.485
Education				
None	**20 (7.4)**	13 (9.9)	7 (5.0)	0,126
Preschool	32 (11.8)	24 (18.2)	8 (5.7)	0.001
Primary school	77 (28.3)	36 (27.3)	41 (29.3)	0.713
High school	**107 (39.3)**	**48 (36.4)**	**59 (42.1)**	0.329
Professional	10 (3.7)	3 (2.3)	7 (5.0)	0.337
Technical	26 (9.6)	8 (6.1)	18 (12.9)	0.065

### Home and Housing Infrastructure

Regarding housing and residence in the area, 81.6% of the surveyed were homeowners with a higher proportion in Corrales de San Luis, Atlántico (92.4%), as well as the time of residence in general was on average 11 (RIC 4–19) years, with a higher average length of stay in Corrales de San Luis, Atlántico (14 RIC 5–25 years). The average household composition was 4 (RIC 3–5) persons; with dwellings that on average had 2 (RIC 2–2) bedrooms and 16.5% overcrowded. Related to basic sanitation, 92.7% of the total population had access to potable water, 99.7% had access to electricity, and only 9% had access to sewerage service. Of the total included, 50.7% had access to propane gas for food preparation and 21.7% used firewood for cooking (see Table 2).

**Table 2:** Household infrastructure of the population studied, Corrales de San Luis, Atlántico and Villanueva (Bolívar)

	Total *N* = 272 n (%)	Corrales San Luis *N* = 132	Villanueva *N* = 140 n (%)	p Value
Own House	222 (81.6)	122 (92,4)	100 (71,4)	< 0,0001
Years of residency Me (RIC)	11 (4–19)	14 (5–25)	7 (3–15)	0,000
Number of people in the dwelling Me (RIC)	4 (3–5)	5 (3–6)	4 (3–5)	0,154
Number of rooms (RIC)	2 (2–2)	2 (2–2)	2 (2–2)	0,708
Overcrowding	45 (16.5)	19 (14,4)	26 (18,6)	0,354
Potable water Me (RIC)	252 (92.7)	127 (96,2)	125 (89,3)	0,029
Tank car	15 (5.5)	3 (2,3)	12 (8,6)	0,023
Water ravine	2 (0.7)	1 (0,8)	2 (0,7)	0.9998
Electric light	271 (99.6)	132 (100)	139 (99,3)	0,331
Sewer	9 (3.3)	4 (3.0)	5 (3,6)	0.803
Food preparation				
Propane gas	138 (50.7)	67 (51)	71(51,2)	0,994
Natural gas	71 (26.1)	7 (5.3)	64 (46.0)	< 0.0001
Firewood	59 (21.7)	57 (43,2)	2 (1,4)	< 0.0001
Electricity	27 (9.9)	23 (17.5)	4 (2.9)	< 0,0001
Coal	1 (0.4)	1 (0.8)	0 (0.0)	0,485
Household roofing material				
Zinc roof tiles	256 (94.1)	121 (91.7)	135 (96.4)	0.095
Wood Cement board Palm	6 (2,2) 5 (1,8) 3 (1.1)	6 (4,6) 1(0,8) 2 (1.5)	0 (0.0) 4 (2,9) 1 (0.7)	0,012 0,371 0,613
Other (Eternit/Plastic)	2 (0.7)	2 (1,5)	0 (0.0)	0,485
Home wall material				
Cement	124 (47.2)	35 (27.6)	89 (65.4)	< 0.0001
Mud	58 (22.1)	55 (43.3)	3 (2.2)	< 0.0001
Wood	58 (22.1)	20 (15.8)	38 (27.9)	0.016
Brick	16 (6.1)	16 (12.6)	0 (0.0)	< 0.0001
Other (Bahareque/Plastic)	7 (2.7)	1 (0.8)	6 (4.4)	0.121
Household floor material				
Cement	156 (57.4)	56 (42.4)	100 (71.4)	< 0.0001
Soil	82 (30.2)	66 (50.0)	16 (11.4)	< 0.0001
Tile	32 (11.8)	8 (6.1)	24 (17.1)	0.004
Wood	2 (0.7)	2 (1.5)	0 (0.0)	0.234
Housing structure				
Independent spaces	250 (91.9)	115 (87.1)	135 (96.4)	0.004
Common area	22 (8.1)	17 (12.9)	5 (3.6)	
Level of household organization				
Organized	237 (87.1)	101 (76.5)	136 (97.1)	< 0.0001
Intradomiciliary disorder	29 (10.7)	25(18.9)	4(2.9)	< 0.0001
Peridomiciliary disorder	6 (2.2)	6 (4.6)	0 (0.0)	0.012
Presence of domestic animals	206 (75.7)	105 (79.5)	101 (72.1)	0.155
Dog	150 (55.1)	82 (53.9)	68 (27.9)	0.025
Birds	77 (28.3)	47 (35.6)	32 (22.8)	0.021
Poultry	69 (25.4)	40 (37.1)	25 (17.9)	0.016
Cat	62 (22.8)	29 (22.0)	22 (15.7)	0.186
None	66 (24.3)	27 (20.5)	39 (27.9)	0.155
Has peridomicile	269 (98.9)	130 (98.5)	139 (99.3)	0.613
Covered	53 (19.5)	32 (24.2)	21 (15)	0.054
Storage	91 (33.5)	26 (19.7)	65 (46.4)	< 0.0001
With domestic animals	162 (59.6)	89 (67.4)	73 (52.1)	0.010

In respect of household infrastructure, 94.5% of the houses had zinc tile roofs, 47.2% of the walls were made of cement followed by 22.1% of mud and wood. Specifically, in Corrales de San Luis Atlántico 43.3% of the homes had mud walls, 50% had cement floors and in Villanueva Bolívar 65.4% of the walls were made of cement and 71.4% had cement floors. Similarly, 91.9% of the households had independent spaces and 87.1% of the households were organized both intra and peri-domiciliary. The presence of domestic animals was found in 75.7%, with dogs predominating in 55.1%, followed by birds in 28.3%. This was related to the presence of 98.9% of peri-domiciliary space, 59.6% of which had domestic animals (see Table 2).

### Knowledge about Chagas Disease (CD)

When asked about the level of knowledge about Chagas disease (CD), 60.7% of the surveyed stated that they had heard of the disease, with a higher proportion (72.9%) in Villanueva, Bolivar (*p* < 0.0001). When asked how CD was transmitted, only 25.4% of the population answered correctly (*p* < 0.003) and 14.7% (p 0.03) of the inhabitants knew some symptom of CD. In this way it was identified that 62.1% (*p* < 0.0001) of the respondents had some degree of knowledge about Chagas disease but when objectifying this knowledge, 86% of the subjects had a very poor level of knowledge about the disease (p 0.024) (See Table 3).

**Table 3:** Knowledge, attitudes and practices related to Chagas Diseases (CD) of population surveyed at Corrales de San Luis, Atlántico and Villanueva (Bolívar), Colombia

	Total *N* = 272 n (%)	Corrales de San Luis *n* = 132 n (%)	Villanueva *n* = 140 n (%)	p Value
A. Knowledge of Chagas disease (CD)				
A1. Has heard of the illness	165 (60.7)	63 (47.8)	102 (72.9)	< 0,0001
A2. Knows how it is transmitted	69 (25.4)	23 (17.4)	46(32.9)	0.003
A3. Knows the symptoms	40 (14.7)	12 (9.1)	28 (20.0)	0.035
A4. Knows the treatment	20 (7.3)	8(6.1)	12(8.6)	0.428
Knows about the disease	169 (62.1)	66 (50.0)	103 (73.6)	< 0,0001
Percentage of knowledge of CD, Me (RIC)	25 (0–50)	12 (0–25)	25 (0–50)	< 0,0001
Very bad	234 (86.0)	120 (91)	114 (81.4)	0.024
Optimum	12 (4.4)	3 (2.3)	9 (6.4)	0.095
Regular	26 (9.6)	9 (6.8)	17 (12.1)	0.136
B. Chagas Disease Vector knowledge				
B1. Recognizes the vector	121 (44.5)	63 (47.7)	58 (41.4)	0.294
B2. Knows where the vector lives	58 (21.3)	37 (28.0)	21 (15.0)	0.008
B3. How the vector is fed	28 (10.3)	23 (17.4)	5 (3.6)	0.000
B4. How the vector is eliminated	40 (14.7)	18 (13.6)	22 (15.7)	0.629
B5. Relationship between domestic animals and Chagas disease	13 (4.8)	9 (6.8)	4 (2.9)	0.126
B6. Presence at home of the CD vector (inside)	34 (12.5)	21 (15.9)	13 (9.3)	0.098
B7. Had been bitten by the vector	9 (3.3)	9 (6.8)	0 (0.0)	0.001
B8. Knows stung people	36 (13.2)	13 (9.9)	23 (16.4)	0.109
Has knowledge of the vector	148 (54.4)	76 (57.6)	72 (51.4)	0.309
Percentage of knowledge of CD vector Me (RIC)	12 (0–25)	12 (0–25)	12 (0.0–12)	0.061
Good	1 (0.4)	1 (0.8)	0 (0.0)	0.485
Bad	11 (4.0)	7 (5.3)	4 (2.9)	0.366
Very bad	255 (93.8)	119 (90.2)	136 (97.1)	0.017
Optimum	0 (0.0)	0 (0.0)	0 (0.0)	--
Regular	5 (1.8)	5 (3.8)	0 (0.0)	0.026
C. Preventive measures of CD vector				
C1. Indoor mosquito netting	62 (22.8)	28 (21.2)	34 (24.3)	0.546
C2. Exterior mosquito netting	11 (4.0)	8 (6.1)	3 (2.14)	0.128
C3. Skin repellent	63 (23.2)	30 (22.7)	33 (23.6)	0.869
C4. Insecticide	167 (61.4)	76 (57.6)	91 (65.0)	0.209

### Vector Knowledge

Investigating the level of knowledge about the triatomine species and with the help of sample images of it, 44.5% of the population stated that they could identify the vector; they also knew the places where the vector was found in 21.3%, and only 10.3% knew correctly how it fed and how was transmitted the infection. It was found that 4.8% of those evaluated knew there was a relationship between the vector, domestic animals and the disease.

Regarding the presence of the vector in the home, 12.5% stated that they had seen it. Therefore, 93.8% (p 0.017) of the investigated subjects were rated regarding their level of knowledge about the vector as very poor (see Table 3).

### Use of Preventive Measures

To avoid contact with the vector, 61.4% of the population uses insecticides, followed by skin repellents (23.2%) and intra-domiciliary mosquito nets (22.8%) (see Table 3).

In an analysis of the population evaluated in the two municipalities and taking into account the degree of knowledge of the disease, it was observed that the female sex had the lowest level of knowledge about the disease and the vector, especially in the age group between 50 and 59 years and in the sector with the lowest level of schooling without discrimination in the occupation developed, which when compared between the two municipalities was statistically significant (See Table 4).

**Table 4:** Knowledge level of Chagas Disease according to Sociodemographic distribution of the population studied, Corrales de San Luis, Atlántico and Villanueva (Bolívar)

	Corrales de San Luis *N* = 132				Villanueva *N* = 140				
	Chagas knowledge		Vector knowledge		Chagas knowledge			Vector knowledge	
	Optimum *N* = 3	Regular/ Bad *N* = 129	Good *N* = 6	Bad *N* = 126	Optimum *N* = 9	Regular/ Bad *N* = 131		Good *N* = 0	Bad *N* = 140
Sex									
F	1 (33.3)	75 (58.1)*	4 (66.7)	72 (57.1)†	6 (66.7)	97 (74.1)		0 (0.0)	103 (73.6)
M	2 (66.7)	54 (41.9)	2 (33.3)	54 (42.9)	3 (33.3)	34 (25.9)		0 (0.0)	37 (26.4)
Age (years)									
1. 0–9	0 (0.0)	8 (6.2)	1 (16.7)	7 (5.6)	0 (0.0)	5 (3.8)		0 (0.0)	5 (3.6)
2. 10–19	1 (33.3)	29 (22.5)	0 (0.0)	30 (23.8)	2 (22.2)	40 (30.5)		0 (0.0)	42 (30.0)
3. 20–29	0 (0.0)	34 (26.4)	2 (33.3)	32 (25.4)	1 (11.1)	32 (24.4)		0 (0.0)	33 (23.6)
4. 30–39	1 (33.3)	22 (17.1)	1 (16.7)	22 (17.5)	2 (22.2)	27 (20.6)		0 (0.0)	29 (20.7)
5. 40–49	1 (33.3)	11 (8.5)	1 (16.7)	11 (8.7)	1 (11.1)	21 (16.0)		0 (0.0)	22 (15.7)
6. 50–59	0 (0.0)	16 (12.4)*	0 (0.0)	16 (12.7) †	3 (33.3)	4 (3.1)		0 (0.0)	7 (5.0)
7. 60–69	0 (0.0)	5 (3.9)	1 (16.7)	4 (3.2)	0 (0.0)	1 (0.8)		0 (0.0)	1 (0.7)
8. 70 o more	0 (0.0)	4 (3.1)	0 (0.0)	4 (3.2)	0 (0.0)	1 (0.8)		0 (0.0)	1 (0.7)
Education									
None	0 (0.0)	0 (0.0)*	0 (0.0)	13 (10.3)	0 (0.0)	7 (5.3)		0 (0.0)	8 (5.7)
Preschool	0 (0.0)	24 (18.6)*	1 (16.7)	23 (18.3) †	0 (0.0)	8 (6.1)		0 (0.0)	41 (29.9)
Primary school	1 (33.3)	35 (27.1)	2 (33.3)	34 (27.0) †	3 (33.3)	38 (29.0)		0 (0.0)	59 (42.3)
High school	1 (33.3)	47 (36.4)	3 (50.0)	45 (35.7) †	3 (33.3)	56 (42.8)		0 (0.0)	7 (5.0)
Professional	1 (33.3)	2 (1.6)	0 (0.0)	3 (2.4) †	1 (11.1)	6 (4.6)		0 (0.0)	18 (12.9)
Technical	0 (0.0)	8 (6.2)	0 (0.0)	8 (6.4) †	2 (22.2)	16 (12.2)		0 (0.0)	0 (0.0)
Postgraduate	0 (0.0)	13 (10.1)*	0 (0.0)	0 (0.0) †	0 (0.0)	0 (0.0)		0 (0.0)	7 (5.0)
Occupation									
1. Agriculture work	0 (0.0)	7 (5.4)*	1 (16.7)	6 (4.8)	0 (0.0)	1 (0.8)		0 (0.0)	1 (0.7)
2. Home	1 (33.3)	53 (41.1)	3 (50.0)	51 (40.5)	3 (33.3)	54 (41.2)		0 (0.0)	57 (40.7)
3. Merchant	0 (0.0)	9 (7.0)	0 (0.0)	9 (7.1)	2 (22.2)	4 (3.1)		0 (0.0)	6 (4.3)
4. Employee	1 (33.3)	7 (5.4)*	0 (0.0)	8 (6.3)	1 (11.1)	19 (14.5)		0 (0.0)	20 (14.3)
5. Student	1 (33.3)	37 (28.7)	1 (16.7)	37 (29.4) †	2 (22.2)	41 (31.3)		0 (0.0)	43 (30.7)
6. Pensionary	0 (0.0)	1 (0.8)	0 (0.0)	1 (0.8)	0 (0.0)	0 (0.0)		0 (0.0)	0 (0.0)
7. Minor	0 (0.0)	2 (1.6)¨*	0 (0.0)	2 (1.6)	0 (0.0)	13 (9.9)		0 (0.0)	1 (0.7)
8. Other	0 (0.0)	14 (10.9)*	1 (16.7)	13 (6.3)	1 (11.1)	12 (9.2)		0 (0.0)	13 (9.3)

* p value < 0,05 compared to regular/bad knowledge of Chagas disease in Villanueva; †, p value < 0,05 compared to bad level of vector knowledge in Villanue-

va; ‡ p value < 0,05 compared to “NO” Covid Risk in Villanueva

Likewise, among the two populations studied, the worst level of knowledge about the disease and the vector was observed in the inhabitants who had lived in their homes for more than 5 years, regardless of the type of construction material (see Table 5).

**Table 5:** Knowledge level of Chagas Disease according to Household structure of the population studied vs. Chagas knowledge in Corrales de San Luis, Atlántico and Villanueva (Bolívar)

	Corrales de San Luis *N* = 132				Villanueva *N* = 140			
	Chagas knowledge		Vector knowledge		Chagas knowledge		Vector knowledge	
	Optimum *N* = 3	Regular/ Bad *N* = 129	Good *N* = 6	Bad *N* = 126	Optimum *N* = 9	Regular/ Bad *N* = 131	Good *N* = 0	Bad *N* = 140
Lenght of residency								
0–1 year	2 (66.7)	8 (6.2)	1 (16.7)	9 (7.1)	2 (22.2)	15 (11.5)	0 (0.0)	17 (12.1)
2–5 years	0 (0.0)	25 (19.4)	1 (16.7)	24 (19.1) †	0 (0.0)	42 (32.1)	0 (0.0)	42 (30.0)
≥ 6 years	1 (33.3)	96 (74.4)*	4 (66.7)	93 (73.8)	7 (77.8)	74 (56.5)	0 (0.0)	81 (57.9)
Roof material								
Palm	0 (0.0)	2 (1.6)	0 (0.0)	2 (1.6)	0 (0.0)	1 (0.8)	0 (0.0)	1 (0.7)
Zinc	3 (100.0)	118 (91.5)	5 (83.3)	116 (92.1)	9 (100)	126 (96.2)	0 (0.0)	135 (96.4)
Cement	0 (0.0)	1 (0.8)	0 (0.0)	1 (0.8)	0 (0.0)	4 (3.1)	0 (0.0)	4 (2.9)
Palm + Mud	0 (0.0)	1 (0.8)	1 (16.7)	0 (0.0)	0 (0.0)	0 (0.0)	0 (0.0)	0 (0.0)
Wood	0 (0.0)	6 (4.6)*	0 (0.0)	6 (4.8)	0 (0.0)	0 (0.0)	0 (0.0)	0 (0.0)
Other (Eternit/Plastic)	0 (0.0)	1 (0.8)	0 (0.0)	1 (0.8)	0 (0.0)	0 (0.0)	0 (0.0)	0 (0.0)
Wall material								
Cement	2 (33.3)	33 (25.6)*	1 (16.7)	34 (28.1) †	6 (66.7)	83 (63.4)	0 (0.0)	89 (65.4)
Mud	0 (0.0)	55 (42.6)*	4 (66.7)	51 (42.2) †	0 (0.0)	3 (2.3)	0 (0.0)	2 (2.2)
Brick	1 (33.3)	15 (11.6)*	1 (16.7)	15 (12.4) †	0 (0.0)	0 (0.0)	0 (0.0)	38 (27.9)
Wood	0 (0.0)	20 (15.5)*	0 (0.0)	20 (16.5) †	3 (33.3)	37 (28.2)	0 (0.0)	0 (0.0)
Cardboard	0 (0.0)	0 (0.0)	0 (0.0)	0 (0.0) †	0 (0.0)	0 (0.0)	0 (0.0)	6 (4.4)
Other (Bahareque/Plastic)	0 (0.0)	1 (0.8)	0 (0.0)	1 (0.8)	2 (22.2)	6 (4.6)	0 (0.0)	0 (0.0)

* p Value < 0,05 compared to knowledge of regular/mal chagas disease in Villanueva; †, p value < 0,05 compared to knowledge of Bad Vector in Villanueva; ‡ p value < 0,05 compared to Covid Risk “No “, in Villanueva

## Discussion

The characterization of knowledge about Chagas disease (CD) in populations with risk factors is essential for prevention strategies like primary and secondary prevention activities aimed for instance to reduce the incidence of new cases, educate the community, and in general, try to control the frequency of the disease not only in populations with high infection rates but also in areas with susceptible communities and elevated probabilities of being infected due to the presence of the vector, the parasite and/or diagnosed patients [8, 9]. This study shows that in most of the surveyed individuals the lack of education related to Chagas and all the basic aspects of the disease is high and in some cases the perception of protection against the illness is wrong, despite of an elevated (apparently) degree of recognition to the illness; this is denoted as many gaps in the accuracy of knowledge in more than 80% of the population included, mostly in persons over 50 years who had the lowest levels of education and restricted healthcare access.

In Latin American countries similar studies and investigations in other regions of Colombia different from ours have findings in this regard. Ruiz et al. in Mexico documented a high nescience of CD in rural populations, with more than 70% of total ignorance about the disease, although this population had a better result in terms of practices related to CD [10]. Similarly, Sanmartino et al. in Argentina probed through KAP strategies that inhabitants of endemic areas have limited knowledge about the Triatominae genus compromising the attitude of the residents towards the presence of this vector in their communities [11]. 

On the other hand, in Colombia, the research performed in this area has controversial results; the KAP surveillance by Cano et al. in Casanare found an adequate degree of understanding about CD in more than 70% of the sample surveyed, and as a result of previous interventions it could be identified the community recognition of several aspects of the Chagas disease [12]. The Colombian (Boyacá) KAP Chagas study published in 2022 by Ramírez Lopez et al. in a different Colombian zone mostly in rural population with similar characteristics like our data, reporting an optimal percentage and level of knowledge but paradoxically with bad practices that did not prevent the disease from being found at home. Our results were that with a high level of knowledge of the disease (most related to transmission) and the vector (recognition), but objectively interpreted in 80% as bad percentage in both (knowledge of the CD and vector) and with satisfactory results of the *“Practice”*component, let us respectfully hypothesize that education strategies, media coverage of some recent cases at this communities, and sometimes the union between the inhabitants could play and important role. Now in the case of both localities (Corrales de San Luis and Villanueva) respect to vector knowledge some differences were found in favor of more recognition of the vector at Corrales de San Luis possible related to the research work done in the past by some universities that included community education strategies about the disease and the vector. Respecting of the cero reports of been bitten by the vector at Villanueva, this could be related with the “Very bad” percentage of vector knowledge witch was 97,1%, suggesting a possible false absent of being bitten by the Triatominae genus in Villanueva. Further studies should be necessary related to this aspect [13, 14]. 

In the case of interventions, Montes et al. in Honduras reported that in exposed populations followed for Chagas disease, a greater degree of knowledge and better practices in relation to the risk of contracting the infection is identified, highlighting also the relevance of health education and the application of prevention strategies at communities focused on train the population at risk against contact with vector and/or the parasite cause of Chagas disease [12, 15]. 

House infrastructure (housing) plays an important role related to vector transmission. A great percentage of the domiciles included were documented to have arranged spaces with low levels of overcrowding but, it was found to had partially connection to basic sanitation services. The insufficient access to sewage and the presence of animals in the peridomicile, predominantly dogs, were noteworthy [16, 17]. Similar investigations have revealed that for example the infrastructure of the dwellings, the construction material as well its internal environment distribution could play a role in favor of the vector reproduction and consequently the prevalence of the infection at risk areas of CD.

Entomological surveys in Central America documented infestation rates > 20%, which is considered high and emphasize the urgency of mitigation strategies. Interventions include housing infrastructure (changing construction materials), especially at the presence of risk factors for infection (vector infestation rate and/or overcrowding spaces and/or high frequency of nearby diagnosed cases) [18]. 

Prevention strategies to control the spread of the vector begins from the basics of parasite, vector and disease knowledge. Therefore, in our research the individuals surveyed revealed that nearly 50% of the sample affirmed to know the vector. Parisi et al. in Bolivia also informed adequate knowledge of the vector, but also found that in consequence of decrease at notification of new infections there was a relaxation of prevention measures and was possible to document a false sense of confidence of the population towards the vector [19, 20]. 

Therefore, the implementation of different strategies and prevention approaches to control the vector requires a multidisciplinary taskforce that includes interventions related to public health, healthcare access (laboratory diagnostic services, clinical updated guidelines) as well as basic sanitation conditions (sustainable development goals 1, 2, 3 6, 11, 13, 15, 17), all this forces together have directly positive impact on the epidemiological indicators of Chagas infection [1, 21, 22]. 

As limitations for this study the research team must declare that a bigger sample and better standardization of the instrument used during surveillance may resolve some doubts.

This original work with non-precedent in these Colombian Caribbean areas may validate the principle that public health intervention strategies in the case of Chagas Diseases should and must be monitored over time uninterruptedly in order to provide updated evidence of surveillance of this pathology that occurs with low frequency or is not reported yet in some Colombian risk areas (Corrales de San Luis and/or Villanueva, Colombia) but has scientific evidence of vector and/or parasite circulation [6, 23, 24]. 

### Electronic Supplementary Material

Below is the link to the electronic supplementary material.

Supplementary Material 1
