# Supplementary material for: Knowledge, Attitudes and Practices of Chagas a Neglected Tropical Disease in Rural Communities of the Colombian Caribbean, CHAGCOV Study
Source: Acta Parasitol. 2024 Apr 9;69(2):1148–56. doi: 10.1007/s11686-024-00833-y (PMC11182857; doi:10.1007/s11686-024-00833-y)
Supplement: Supplementary file 1 — Supplementary Material 1 [file 11686_2024_833_MOESM1_ESM.docx]

**Knowledge, attitudes and practices of Chagas a Neglected Tropical Disease in rural communities of the Colombian Caribbean, CHAGCOV study.**

Margarita M Ochoa-Diaz^1,2, ¶^, Daniela Orozco-Garcia^1,2, ¶^, Ronald S. Fernandez-Vasquez^1,2, ¶^, Melisa Eyes-Escalante^3,4, ¶^

^1^ School of Medicine, Universidad del Sinú Seccional Cartagena, Cartagena, Colombia.

^2^ Research Group GIBACUS, Tropical Medicine.

^3^ School of Biology, Tropical Medicine Doctorate, Universidad del Atlántico, Barranquilla, Colombia

^4^ Research Group Biodiversidad del Caribe Colombiano

Address: School of Medicine, Universidad del Sinú, seccional Cartagena, 130001, Cartagena, Colombia

Corresponding author: [mdochoadiaz@gmail.com](mailto:mdochoadiaz@gmail.com)

^¶^ These authors contributed equally to this work.

**Keywords:** Chagas disease, Neglected Tropical Disease, Sustainable Development Goals, Colombia

**Statements and Declarations:**

**Conflict of interest:** The authors declare no competing interests.
